# Dance for people with chronic breathlessness: a transdisciplinary approach to intervention development

**DOI:** 10.1136/bmjresp-2020-000696

**Published:** 2020-11-09

**Authors:** Samantha Harrison, Krzysztof Bierski, Naomi Burn, Sarah Mclusky, Victoria McFaull, Andrew Russell, Gaynor Williams, Siân Williams, Jane Macnaughton

**Affiliations:** 1 School of Health and Life Sciences, Teesside University, Middlesbrough, North Yorkshire, UK; 2 Department of Anthropology, Durham University, Durham, UK; 3 Institute for Medical Humanities, Durham University, Durham, UK; 4 Corporate Personal Fitness, Middlesbrough, UK; 5 Breathe Easy, Darlington, UK; 6 London Respiratory Network, Strategic Clinical Networks, NHS England (London Region), London, UK

**Keywords:** exercise, pulmonary rehabilitation

## Abstract

**Objectives:**

A transdisciplinary research approach was used to develop a holistic understanding of the physical and psychosocial benefits of dance as an intervention for people living with chronic breathlessness.

**Methods:**

The dance programme was developed in collaboration with British Lung Foundation Breathe Easy members in NE England (Darlington) and London (Haringey). Members of the Darlington group were invited to participate in the programme. An exercise instructor, trained and mentored by a dance facilitator delivered 60–90 min dance classes for 10 consecutive weeks. Exercise capacity, mobility, quadriceps strength, health status, mood and interoceptive awareness were assessed at baseline and after the 10-week programme. Second-to-second heart rate (HR) monitoring was conducted during one of the classes.

**Results:**

Ten individuals were enrolled (n=8 women). Mean (SD) age was 70 (24); Body Mass Index 29.7 (8.1) kg/m^2^; one participant used oxygen and one a walking aid. Seven completed the dance programme. Improvements in all outcome measures were detected, with the exception of the Multidimensional Assessment of Interoceptive Awareness, which individuals found hard to comprehend. Eight participants wore HR monitors during one dance class and spent on average 43.5 (21.8) min with HR corresponding to at least moderate intensity physical activity (≥64% HRmax). People found the dance classes enjoyable and those with relevant past experiences who are optimistic, committed to staying well and playful readily adopted the programme.

**Conclusion:**

A dance programme bringing both physical and psychosocial benefits for people with chronic breathlessness is acceptable when coproduced and evaluated through a transdisciplinary approach.

**Key messages**

- Engagement with pulmonary rehabilitation among people living with chronic breathlessness is frequently poor.
- A coproduced dance programme that is aligned with people’s previous experience, culture and values might have greater uptake and better sustainability.
- This transdisciplinary pilot study suggests a full-scale randomised controlled trial of such a programme would be worthwhile, and reports on the acceptability of the research measures to participants.

## Introduction

Interventions to promote daily physical activity are important in the management of patients with chronic breathlessness,^1^ not least because low levels of physical activity are associated with an increased risk of hospitalisation and mortality.^2^

Pulmonary rehabilitation (PR) is recommended for all individuals with symptoms of breathlessness, yet engagement is often poor and improvements in exercise tolerance do not translate into meaningful gains in physical activity away from the PR setting.^3 4^ There are likely a number of complex and individualised reasons for this. In part, the language used to describe PR and the unfamiliarity of gym-like spaces may deter people from attending^5^ and absence of choice in PR programmes (the majority offer either walking or circuit-based exercise) may limit the transferability of culturally appropriate activities into people’s everyday lives. Aligning exercise programmes with activities that are valued, enjoyed and considered to be meaningful by the patient population may help promote uptake and encourage sustainable behaviour change.^6^

One such activity, aligned with values, previous experience and culture, is dance. Dance programmes have been found to be beneficial for older adults, those at risk of falls and people with neurological conditions including Parkinson’s disease.^7–9^ They could be a useful adjunct to formal PR for some people, but research is required to provide evidence for this. To date, the only published study examining the feasibility of a dance intervention for people with lung disease has been conducted in Toronto, Canada^10^ although research in London, UK is also underway. The authors of the Toronto study reported that the delivery of 60-minute dance classes, two times a week for 8 weeks, was feasible (49% uptake and 95% completion rate) and safe.^10^ Improvements in exercise tolerance, balance, balance confidence and health status were observed but did not reach clinical significance. There were no improvements in daily step count, although the small sample size (n=20) and large variability in daily steps may account for this. It could be that like PR, dance does not translate into improved physical activity away from the intervention setting but there may be other advantages besides this, such as psychological gains associated with group solidarity, friendship and fun. A recent evaluation of the ‘Dance to Health’ programme at Sheffield Hallam University, initiated to reduce falls in older people, reported that the main appeal of dance was ‘meeting new people’ and 87% said they made new friends as a result of participating.^9^

Understanding the benefits and how they are conferred can help inform and optimise intervention delivery. On an individual level, dance may increase interoceptive awareness (body awareness), making it particularly appropriate for a population of people with chronic breathlessness. Collaborative work within our project with neuroscientists suggested that people with chronic breathlessness have poor interoceptive awareness, partly because of comorbidities such as anxiety and depression.^11^ This may account for the discordance between symptoms experienced and lung function observed in people with Chronic Obstructive Pulmonary Disease (COPD). If interoception can be enhanced, by drawing awareness to the body as having the potential to provide enjoyable movement rather than ‘exercise’, then symptom management may also be improved, leading to a greater willingness to engage in activities that provoke breathless symptoms.^12^

The aim of this paper, therefore, is to use a transdisciplinary research approach^13^ to develop a holistic understanding of dance as a physical activity intervention for people living with chronic breathlessness. Specifically, we will report (1) *Decision-making processes*; preparatory work informing the content of a tailored dance programme for individuals living with chronic breathlessness in the North East of England, (2) *Methods and findings*; the content/delivery of dance and information gleaned from functional tests, self-reported questionnaires, heart rate (HR) monitoring and anthropological methods, (3) *Feasibility elements*; the acceptability of dance, adherence rates and willingness/ability to complete outcome measures.^14^ This information will inform the delivery of a sustainable community dance programme and a future definitive research trial.

## Methods

### Study design

We adopted a transdisciplinary approach to intervention development and conducted elements of feasibility testing.^14^ An equal status, simultaneous mixed methods study design was applied.

### Preparatory work informing decisions

The idea to initiate a dance programme was conceived by researchers on the Wellcome-funded *Life of Breath* (LoB) project working with SW, who was already providing dance classes for British Lung Foundation Breathe Easy members in London (Haringey). Work by the LoB research team had identified three reasons why dance might be an effective intervention. First, that the language of dance put up fewer barriers than ‘PR’. Previous work had demonstrated that participants were embarrassed by the notion that they were attending ‘rehabilitation’, and even the clinical language of ‘pulmonary’ was off-putting.^5^ Second, dance, its language, personal expression and rhythms, was part of the life experience of our intended participants in the North East of England (Darlington), whereas a gym culture was not. Third, dance activity, involving awareness of the whole body, might improve interoceptive awareness.^12^

### Patient and public involvement

The LoB project had already established strong links with ‘experts by experience’ in the Breathe Easy Darlington (BED) group, led by Gaynor Williams. The dance class in London was filmed and shared with BED members along with participants’ personal accounts of participating in dance.^15^ A project Steering Group was established. Meetings and agendas were set by the LoB team and it was co-chaired by JM and GW (ie, a researcher and a participant). Meetings took place once a month and involved researchers from LoB, lead members of BED, SW (dance facilitator) and VM (an exercise therapist already working with BED). Discussion focused on the practicalities and sustainability of the programme and also on how best to disseminate and publicise the findings. BED members were fully involved in planning and delivery of the programme, in ensuring the acceptability of the research methods used including what data might be collected and how long this would take. For example, researchers did not collect information on specific lung conditions as the participants preferred the focus to be on the symptom of breathlessness rather than on underlying condition. Participants’ major concern was continuity of the programme following the research project. To ensure this, SW trained VM in dance instruction.

### The dance programme

The dance programme was advertised among BED members by word of mouth. Membership in BE support groups is available for people who have a lung condition and experience symptoms of breathlessness and their carers. Any member of BED who was not a carer was eligible to take part. Sixty to 90-minute dance classes were offered weekly for 10 consecutive weeks. They were delivered by VM, with no assistant; SW attended two sessions to offer support. VM danced with the group, demonstrating the dances in advance where necessary, but otherwise inviting participants to follow along. Beginning with a 10-minute warm-up, which started with circling movements for joint mobilisation, then chi gung movements to music, the classes consisted of 2–3 minute standing and sitting dances with short rest periods in between followed by a 10-minute cool down and stretch. The focus in all the dances was on step patterns rather than arm movement, in line with PR targets. These were inspired by social dances from the 1950s to today, line dancing and English country dancing that some participants recognised from different phases of their life and are easy to learn. Fast turns and spins were not included as they can cause people to overbalance, but gentle quarter and half turns added variety to repeated sequences. Twisting motions were optional, depending on people’s joint health. To encourage hip stability, side steps were incorporated, for example, in a circle dance. Circle dances have the advantage of working in a limited space and enable connection between dancers without partner dancing that can be off-putting for some. Dancers each held resistance bands between them rather than hands, which can feel too intimate. One dance was inspired by the participants who held an anniversary party where they danced and kept going despite their breathlessness by holding onto chairs and tables; this became a new standing chair dance with a powerful new story. Participants would always start the class from a sitting position taking a couple of minutes to rest. Each dance was followed by a short pause and participants would almost always sit down before resuming movement. Between six to eight dances were performed during each session. The music selection was original not traditional and based on tempo and rhythm; slow enough for participants to enjoy dancing and keep in time; with a strong enough beat to enable quick learning of the steps. It included different genres including Rock, Motown, Northern Soul and classical.

### Data collection

The exercise instructor recorded attendance at each dance class. Demographic and anthropometric information including age, sex, Body Mass Index (BMI), oxygen and gait aid usage were recorded at visit 0 (baseline) and at visit 1 (10-week follow-up). Most of the participants had prior experience of dance, including ballroom, line dancing and ballet as well as movement practices such as Tai Chi and Taekwando. Exercise capacity, mobility, quadriceps strength, health status, mood and interoceptive awareness were assessed at baseline and after the 10-week programme.

*The six-minute walk test (6MWT)* is a valid, reliable and responsive measure of functional exercise capacity. It was conducted according to international recommendations^16^ and the total distance walked was recorded in metres. The minimal clinically important difference (MCID) for the 6MWT in people with COPD is 25–35m.^17 18^

*The Timed Up and Go (TUG) test*^19^ provides a timed measure of functional mobility. The test requires individuals to stand from a chair, walk 3 m at a comfortable pace and return to a sitting position. The time taken is recorded in seconds. A TUG value longer than 11 s has been shown to predict falls in people with COPD.^20^

*The 30-Second Sit to Stand Test (30-STS)* requires participants to stand from a chair and sit fully, completing as many repetitions as possible in 30 s. It is a valid and reliable measure of quadriceps strength.^21^ The MCID has been quoted as ≥2 pre–post PR.^22^

*The COPD Assessment Tool (CAT)* is an 8-item measure quantifying the impact of COPD symptoms on health status.^23^ Scores range from 0 to 40 with higher scores indicating a greater impact on health status. It has good psychometric properties and is responsive to PR and the MCID is estimated to be −2 points.^24^

*The Patient Health Questionnaire-9 (PHQ-9)*^25^ and the Generalised Anxiety Disorder Assessment-7 (GAD-7)^26^ assess symptoms of depression and anxiety respectively in the previous 2 weeks. Items are rated on a 4-point scale with higher scores indicating greater severity of psychological symptoms.

*The Multidimensional Assessment of Interoceptive Awareness (MAIA)*^27^ is a 32-item state-trait questionnaire collecting information on body awareness. It comprises eight scales: noticing, not distracting, not worrying, attention regulation, emotional awareness, self-regulation, body listening and trusting. Items are rated on a 6-point scale with higher scores indicating higher positive awareness.

*HR monitoring* was conducted second-to-second during a single dance class (duration ~90 min) using wrist-worn monitors (Polar A360, Polar Electro, Kempele, Finland). Age predicted maximal HR (HR_max_) was calculated for each participant using the Tanaka equation (208−0.7×age in years)^28^ or a modified HR_max_ equation (164−0.7×age in years) for participants on beta-blockers or medication affecting HR.^29^ If a participant exceeded this predicted value during the dance class, their HR_max_ was amended to the higher observed value.^30^ Expressed as a percentage of the participants’ HR_max_ mean HR during the dance class was calculated and the highest one-second value during the dance class was taken as the peak HR. Time spent with HR corresponding to at least moderate intensity physical activity was calculated (eg, ≥64 % HR_max_).^31^ Exertional dyspnoea was assessed before and after the dance class using the modified Borg Dyspnoea Scale and the rating of perceived exertion scale.^32 33^

*A qualitative component* involved a researcher (KB) working in the dance classes as a participant–observer focusing on process issues such as how participants engaged with the choreography, how they overcame social inhibitions and personal/illness difficulties and how they commented on their changing abilities and breathing patterns. Informal group discussions led by KB involving all participants were held following each class. Open questioning with more focused follow-up provided insight into learning strategies, personal experiences of dance, bodily awareness and factors that might prevent or facilitate sustainability of dance practice. The group discussions were audio-recorded and included alongside detailed field notes. The researcher also joined informal social gatherings of the group held before each class as well as occasional group events such as informal walks and the 10-year celebration of the group’s formation. KB’s presence at these activities was as an observer and he was interested to note that dance featured centrally as a fun activity at the celebration.

### Analysis

Descriptive data (median IQR) are displayed for the 6MWT, TUG, 30-STS, CAT, PHQ-9, GAD-7 and the individual subscales of the MAIA at the two time points; pre and post the dance programme. Inferential statistics were not performed due to the small sample size. All HR data are presented as mean (SD). The analysis of the anthropological material proceeded from field notes and recorded group discussions. These data were transcribed and recurrent themes identified through a grounded theory approach and manually coded by KB. Themes and conclusions were compiled in a qualitative report that was shared with and confirmed by the research team.

## Results

The dance programme was offered to 24 members of BED. Reasons for not wishing to take up dance included: lack of interest in dance/thinking they cannot dance, work/child care commitments and fear of breathlessness/activity. Ten individuals were enrolled in the dance programme (n=8 women; mean (SD) age 70 (24) years; BMI 29.7 (8.1) kg/m^2^, baseline Borg breathless rating 1 (2) arbitrary units, n=1 oxygen usage and n=1 walking aid).

The 6MWT, TUG, 30-STS, CAT, PHQ-9, GAD-7 and MAIA were completed before and after the 10-week dance programme. The results are displayed in table 1.

**Table 1:** Outcome variables pre and post the dance programme

Variable	Pre(median (IQR))	Post(median (IQR))
6MWT (min)	280.5 (254.5–312.5)	307.1 (82.1) 291.0 (263.5–321.0)
TUG (s)	10.8 (8.7–12.7)	10.0 (7.0–11.0)
30-STS	10.0 (9.0–12.3)	13.0 (11.0–14.5)
CAT	27.5 (23.0–32.0)	27.0 (25.0–28.5)
PHQ-9	14.5 (5.0–17.0)	8.0 (4.5–10.5)
GAD-7	5.0 (1.8–12.0)	5.0 (0.5–6.0)
MAIA: noticing	5.2 (3.9–5.9)	3.7 (3.2–5.0)
MAIA: not distracting	1.5 (0.7–2.0)	1.3 (1.3–1.7)
MAIA: not worrying	2.5 (2.3–2.9)	2.7 (2.2–3.7)
MAIA: attention regulation	3.0 (2.4–3.4)	2.7 (2.3–3.2)
MAIA: emotional awareness	3.6 (3.3–3.8)	3.6 (3.4–4.2)
MAIA: self-regulation	3.3 (2.8–3.7)	3.5 (3.1–3.6)
MAIA: body listening	2.3 (1.0–3.0)	2.3 (1.7–3.3)
MAIA: trusting	3.0 (2.3–3.2)	3.3 (3.0–3.8)

CAT, COPD assessment tool; GAD-7, Generalised Anxiety Disorder Assessment; MAIA, Multidimensional Assessment of Interoceptive Awareness; 6MWT, six-minute walk test; PHQ-9, Patient Health Questionnaire; 30-STS, 30 s sit to stand test; TUG, timed up and go.

Eight participants wore HR monitors during a single dance class (n=7 women, mean (SD) age 68.5 (7.3) years; BMI 31.2 (9.2) kg/m^2^, n=1 oxygen usage and n=0 walking aid). One monitor was faulty and did not record, meaning data were available for n=7. One individual was in addition to those who had completed the functional tests/questionnaires. Three individuals were on beta-blockers or medication affecting HR. The mean (SD) HR throughout the dance session corresponded to 65 (6)% HR_max_ and peak HR was achieved during the dance session corresponded to 92 (10)% HR_max_. Participants spent on average 43.5 (21.8) minutes with HR corresponding to at least moderate intensity physical activity (eg, ≥64 %HR_max_). Baseline Borg rating was 2 (1.1) arbitrary units (AU) (‘slight’) and postsession Borg was 3 (1.2) AU ‘moderate’. Postsession Rate of Perceived Exertion (RPE) was 12 (2.2) AU ‘fairly light’ to ‘somewhat hard’.

Key themes derived from the qualitative findings were bonding among the group, the sense of learning and being together, fun and a feeling that they were not ‘exercising’, the impact of dance itself and a common commitment among participants to staying well. One participant commented on the bonds formed between members of the group through the shared experience of dance:

*It’s the social side of it. I like to do the dances, everybody is nice, everybody knows everybody*.

It was clear that a shared sense of background, place and culture contributed this sense of connection. Despite people from the Haringey group being of a similar age and living with chronic breathlessness, their social/cultural circumstances were viewed as being very different. The BED group compared themselves to the London group:

Having been to Haringey and seen what they do there, I noticed we’re different, we’re different people.

A sincere sense of learning, developing together and having fun were expressed rather than striving to achieve a goal, contrasting with the individualistic and outcome focus of PR:

Today’s class was good, we all got up and we all participated and I think we all did well.

We laugh, even though I am terribly out of breath today, still enjoyed it, and we’ve laughed at each other and laughed at ourselves.

Dance was described as fun and conducive to therapeutic laughter distracting from the experience of undertaking exercise:

The dance is enjoyable that’s the reason. We all laugh. It’s not sort of exercise.

I prefer this [dance] to exercise. There’s something about the movements that seem to be more sedate and strengthening.

The movements undertaken during the dance sessions seemed to contribute to a holistic sense of well-being, by taking participants out of themselves, enabling them to forget they were ill and by giving them the feeling of improved function, even when the quantitative measures did not necessarily bear this out:

When I’m dancing, I’m no longer myself. I’m my right and my left, I’m my movement… I forget I’m ill.

We can concentrate on our dancing and not on our breathing.

My balance wasn’t very good to start with and I feel not that it got much better.

I just get so much more from this. I’ve noticed definition on my muscles on my arms and legs.

Each week I’m looking forward to dancing on Friday.

Motivation to participate in dance stemmed from previous positive experiences, ‘It’s really nice to be back dancing’, which often provoked sentimental feelings of nostalgia. Those who chose to attend the dance classes were generally optimistic, committed to staying well and voiced a playful approach:

I feel like a kid in the playground again.

It’s playful, it just relaxes you.

Of the 10 people enrolled into the programme, 7 completed (70%) (defined as attending the follow-up assessment), 3 withdrew; one was unwell, one felt embarrassed due to use of oxygen prohibiting engagement and one had difficulties with the group dynamics. The median (range) number of classes attended was 8 (6–10). Although we chose to run the programme in the spring and early summer, usually the best time for people with respiratory conditions, the main reasons why classes were missed were because of illness and caring responsibilities. There were no adverse events during the dance classes. All participants enrolled completed the outcome measures at baseline and all seven completers repeated these following the dance programme. However, most reported dissatisfaction with the MAIA, finding the questions repetitive and difficult to interpret (‘I just don’t think like that’). This reflects other work by the LoB team with patients who found the Multidisciplinary Dyspnoea Profile questionnaire similarly problematic.^12 34^ Finally, the exercise instructor delivered all the dance classes and the participants reported building a strong rapport with her that reflected feelings of trust and safety. It is highly likely these dance activities will continue when circumstances permit.

## Discussion

Adopting a transdisciplinary approach we have developed a dance programme as a physical activity intervention for people living with chronic breathlessness. Dance was acceptable to individuals living in the North East of England and adherence rates were high (70%). An exercise instructor, trusted by the participants, with training and mentorship from a dance facilitator, successfully delivered the 10-week dance programme. Full engagement between researchers and participants was an important part of this study. In keeping with this, the monthly steering group, supported also by weekly calls between dance facilitator and instructor, ensured that the programme incorporated the needs of the group, their stories, music choices and movement needs and enabled us to establish trusting relationships across the project team. The outcome measures were feasible to complete although some issues with the measure of interoception were detected. Participation in a dance class corresponded to approximately 44 minutes participation in at least moderate physical activity, while only incurring moderate symptoms of breathlessness. People found the classes enjoyable and those with relevant past experiences who are optimistic, committed to staying well and playful, readily adopted the dance programme.

Videos and accounts from peers were used to encourage uptake of the dance programme, however, acceptance rates were similar (42%) to those observed in PR.^35^ Similar attempts to encourage uptake to PR have had limited success, for example, a co-designed education video or the offer of a ‘taster session’ did not improve uptake of PR following an acute exacerbation of COPD.^36 37^ Although people receiving peer support often place high value on a ‘living example’ of someone experiencing the same challenges, it is important that patients are able to relate to peers,^38^ and the qualitative results suggested that the BED group saw themselves as very different from the Haringey group. Part of this related to how well they were able to carry out the dance moves at first, but there is a deeper reason that relates to the way in which dance is culturally located within region, memories and experiences of the participants. This is a common thread in many arts-based interventions, such as singing,^39^ and signals the importance of fully involving participants in the organisation and design of such programmes to ensure engagement. Since completion of the research study two more members have joined the dance classes, with no further input from the researchers, and one person who dropped out (due to difficulties using oxygen) has since returned. No participants have dropped out over the 5-month period between study completion and the programme being forced to stop due to the outbreak of COVID-19. It seems likely that uptake to the dance programme will snowball as other members enrol based on the positive accounts provided by their peers whom they know and trust.

Although acceptance was initially lower than hoped for, attendance and completion rates were high (70%). Allowing participants to select their own music that was culturally relevant endorsed the feelings of ownership, already signalled by the collaborative steering group. Such feelings are necessary for behaviour change and enabled moments of nostalgia connected to positive experiences and emotions. The exercise instructor was known to many of the participants and was someone they trusted and with whom they felt safe, feelings that are particularly important when engaging in an intervention that can promote breathlessness, a symptom associated with increased anxiety and fear of dying.^40^

People with COPD and depression are less likely to attend PR. We might expect those who choose to engage in a dance programme to have better mood. This is true of those enrolled in a feasibility study of dance conducted in Canada who, at baseline, had no presence of anxious or depressed symptoms.^35 41^ However, the members of BED enrolled in this study did present with moderate symptoms of anxiety and depression, which reduced to mild symptoms following completion of the programme, supporting the view that dance can have a positive impact on people’s mood and emotional state. There are a number of likely reasons for the positive impact of dance on mood. In part, it may be due to functional gains obtained through participation in choreographed dances that require shifting weight, turning and dual tasks (eg, remembering choreography while moving to the beat). However, the enjoyment felt during the dance classes and the social connectedness observed by the participant-observer and described by the participants is likely to have had a significant role in improving mood and encouraging attendance/completion.

Gains in physical function, including improved exercise capacity, strength and balance, are likely training effects obtained via people’s participation in dance-based activity for approximately 44 minutes at 64% of their HR_max_, which is the recommended intensity to elicit improvements in physical fitness,^42^ yet symptom perception was low. Despite engaging in moderate intensity activity, people did not perceive being very breathless or exerting themselves hard. Participants’ comments suggest that this is because the enjoyment of dance deters acknowledgement of unpleasant bodily sensations such as breathlessness. Another explanation would be that through participation in dance, participants have developed a better sense of interoception, translating to better awareness and thus management of symptoms. However, no change in interoception was detected using the MAIA tool, although its cultural specificities (it was developed in the USA in a predominately middle-class population) were commented on by several participants. The choice of instructor was led by the Breathe Easy Group who wished their emotional and financial investment in this new programme to have the potential for continuity. Until the advent of COVID-19, this was in fact taking place with the BED group making the decision to pay for regular weekly dance classes, with an expanded participant group.

This study was conducted in the North East of England and culturally specific differences regarding willingness to engage in dance and music preferences exist between regions and countries, potentially limiting the generalisability of these findings. By targeting a Breathe Easy support group it is likely we were recruiting from a pool of motivated and conscientious individuals as they had already independently sought support. By the nature of self-selection it is perhaps unsurprising we gleaned positive responses regarding participation in dance as those who were not interested in dance did not select to take part. Due to between-subject variation in true HR_max_^28 29^ the use of prediction equations to estimate participants HR_max_ could also be considered a limitation of this study. This may have led to overestimation or underestimation of the true exercise intensity of the dance class and the number of minutes spent with HR corresponding to at least moderate intensity physical activity. Furthermore, the majority of participants had been participating in an exercise programme led by the same instructor (‘BeActive’) consisting of circuit training two times a week prior to enrolment in the dance programme. They also continued to attend BeActive once a week while participating in the weekly dance classes. It is unlikely that any of the functional gains observed were due to participation in BeActive as all had been undergoing the programme for a long period of time. Instead we may have underestimated the effects of the dance; a group naive to exercise may have had greater benefits. That said, in the absence of a control group, we cannot discount that the interaction between the two programmes (Dance and BeActive) resulted in functional gains.

In an age of social distancing, especially for vulnerable groups like those with respiratory problems, the kinds of social benefits, such as peer support, encouragement and laughter, that were noted in our study, may be difficult to replicate online. The LoB project has created video material for individuals to engage in dance in their own homes and it is hoped this might span the gap until these classes can start up again.^15^

## Conclusion

Our study demonstrates that a dance programme, as a physical activity intervention, is acceptable to individuals with chronic breathlessness living in the North East of England. Our transdisciplinary research approach, which involved both quantitative and qualitative assessment as well as coproduction of the programme with our ‘experience’ partners in BED, was critical to establishing the intervention, exploring its benefits and ensuring its sustainability. Future research in the form of a pilot randomised controlled trial is now planned to examine the role of dance as a way of addressing physical activity in people with COPD.
